# Evaluating the Impact of Telehealth-Based, Diabetes Medication Training for Community Health Workers on Glycemic Control

**DOI:** 10.3390/jpm10030121

**Published:** 2020-09-11

**Authors:** Casey N. Keegan, Craig A. Johnston, Victor J. Cardenas, Elizabeth M. Vaughan

**Affiliations:** 1 School of Health Professions, Baylor College of Medicine, Houston, TX 77030, USA; caseykeegan1010@gmail.com; 2 Department of Health and Human Performance, University of Houston, Houston, TX 77004, USA; cajohn25@central.uh.edu; 3 Department of Internal Medicine, University of Texas Medical Branch, Galveston, TX 77555, USA; vcardena@utmb.edu; 4 Department of Medicine, Baylor College of Medicine, Houston, TX 77030, USA

**Keywords:** education, telemedicine, Community Health Workers, diabetes, group visits

## Abstract

Background: Diabetes is a major contributor to morbidity and mortality. Community Health Workers (CHWs) have been instrumental in improving patient outcomes. However, CHW training largely focuses on general diabetes concepts rather than medications. Providing accessible, diabetes medication training for CHWs has the potential to increase patient understanding, personalized care, and adherence, thereby improving outcomes. Objective: To evaluate the impact of a telehealth-based diabetes medication training for CHWs on patient outcomes as measured by HbA1c changes. Methods: We provided a 12-month weekly, telehealth (videoconference) medication training for CHWs who led 6-month diabetes programs for low-income Latino(a)s in community clinics. We measured participant HbA1c (primary outcome), blood pressure, and body mass index (BMI) changes. We evaluated CHW knowledge via two pre/post-tests: medication adverse events/side effects (TEST-1, months 1–6) and dosing, titration, and emergencies (TEST-2, months 7–12). We assessed CHW training application by their ability to identify patient, provider, and healthcare system medication barriers. Results: Participants’ (n = 55) HbA1c improved (9.0% (75 mmol/mol) to 7.8% (62 mmol/mol) (*p* = 0.001)). Blood pressure and BMI changes were not significant. CHWs improved their knowledge: TEST-1: 10.5-18.2/20.0 (*p* = 0.002), TEST-2: 10.3–17.3/19.0 (*p* = 0.0019). CHWs identified 984 patients (n = 610), providers (n = 151), and healthcare systems (n = 223) medication barriers during the 12-month training. Conclusions: Providing a telehealth-based, diabetes medication training program for CHWs allowed a personalized approach to identify barriers to care at several levels, which was associated with significant participant HbA1c reductions and improved CHW knowledge. This is a promising cost-effective, culturally sensitive strategy to improve diabetes care. Larger longitudinal evaluations are needed to fully understand the impact of CHW medication training.

## 1. Introduction

A total of 425 million people are living with diabetes worldwide [1]. In the United States, diabetes is the seventh leading cause of death, and there are 1.5 million new cases each year [2]. Low-income and minority populations are twice as likely to be diagnosed with diabetes and have 50% higher mortality rates [3]. Nearly half (45%) of individuals with the disease do not achieve target HbA1c levels, which has been closely associated with medication nonadherence [4,5,6]. Community Health Workers (CHWs), or *promotores de salud*, are local individuals who assist in connecting the healthcare system to local residents and have been an integral part of caring for low-income and minority populations [7,8]. Their insight in the social determinants of health uniquely positions them to identify and overcome healthcare barriers in culturally specific ways [9,10]. However, CHW training largely addresses general disease concepts or skills, e.g., diabetes self-management and not medication education [11,12,13].

Medications are vital pieces in patient care and achieving glycemic control [4,5,6]. Though medication adherence is often be assumed to be primarily patient-related, it is multifaceted and complex [4]. In addition to patient factors (e.g., dosing or titration misunderstandings, poor treatment plan involvement, insufficient transportation), adherence relates to providers (e.g., complex or high-cost regimens) and healthcare systems (e.g., eligibility, affordable care, refill processes) [4,6]. Perceived treatment ineffectiveness is a major determinant, whereas objective improvements (e.g., reduced HbA1c levels) increase adherence [5]. Strategies to improve adherence are similarly complicated, requiring strong and ongoing communication at all three levels. This is a challenging task universally but particularly in resource-limited, culturally diverse populations [6].

Diabetes medication training for CHWs has great potential for individualized therapy for vulnerable populations [4,14,15,16]. Specifically, this personalized approach has the capability to identify each individual’s barriers to care in addition to addressing misbeliefs, distress, and trust [4,14,15,16,17]. It is critical to provide accessible training for CHWs in order to overcome logistical barriers including time constraints and transportation to the site [18]. We previously demonstrated the feasibility and acceptability of achieving robust CHW training via telehealth [13]. We also showed that a multifaceted diabetes program for low-income individuals is valuable for diabetes outcomes [19]. However, two major components that have not been evaluated are the ability to train CHWs on diabetes medications and the effect of this training on patient outcomes, which may be paramount in healthcare for underserved individuals.

The objective of this study was to evaluate the impact of a 12-month, telehealth medication training for CHWs who led 6-month diabetes programs for low-income Latino(a)s with type 2 diabetes at community clinics [19]. Our outcomes included participant HbA1c (primary outcome), blood pressure, and body mass index (BMI) changes, evaluation of CHW knowledge via two pre-/post-tests, and training application as measured by the CHWs’ ability to identify patient, provider, and healthcare system medication barriers. We hypothesized that this personalized approach would result in improved participant outcomes, specifically HbA1c levels, and that CHWs would increase their medication knowledge and demonstrate application of their training.

## 2. Methods

### 2.1. Study Design

This study evaluated the impact of telehealth-based diabetes medication training for CHWs on participant HbA1c levels at an urban community clinic in Houston, Texas. This study occurred from July 2018 to June 2019. The Institutional Review Board at Baylor College of Medicine approved the study (H-40322).

The intervention provided weekly (months 1–6) or bimonthly (months 7–12) CHW mobile health (mHealth) contact. The intervention also included CHW-led diabetes group visits that were conducted in Spanish and held monthly for 6 months. Group visits consisted of large and small group education, a 1:1 provider visit for medication management, and a healthy group meal. CHWs were assigned 3–5 participants to contact via mHealth (phone or text) for coaching, education, and to relay concerns (i.e., refills, adherence) [19]. CHWs maintained participant reports of their interaction and reported these to study physicians weekly, except for acute issues (e.g., hypo-/hyperglycemia) that were immediately addressed. The study design was based on the clinical trial, *T*elehealth-support, Integrated care with CHWs, *ME*dication-access (TIME), which occurred from January 2018 to December 2018 [19].

Participants were identified using a clinic database that searched for adult Hispanics/Latino(a)s with type 2 diabetes (ICD E11.X). Study eligibility included: low-income (≤250% United States federal poverty level [20]), ≥18 years-old, Latino(a)s, ability to understand Spanish, and diagnosed with type 2 diabetes (e.g., HbA1c 6.5% (48 mmol/mol) or higher, a physician-documentation of the disease in their medical record). Participants were excluded if they were not appropriate to treat in a group setting, e.g., needed more or frequent individualized physician attention, or did not attend at least one group visit. Participants provided written consent and confidentiality statements. CHWs were Latino(a), Spanish-speaking or bilingual, and maintained an active Texas CHW certification [21]. CHWs lived in low-income areas in Houston, Texas and were recruited from local clinics, churches, or by word of mouth. CHW and participant recruitment strategies were consistent with our prior work [13,19].

Community Health Worker telehealth training occurred for one hour/week during the 12-months and consisted of support (participant-related questions or concerns; 30-min) and training (medication adverse events, dosing, titration, emergencies; 30-min). CHWs received an updated participant medication list from study providers no less than every month. CHWs received iPads to participate in the video conference (ZOOM) trainings. ZOOM is an encrypted system that is efficient in low-bandwidth settings [22]. CHWs used their iPads or a cellular device for mHealth communication with participants and study physicians via OhMD, a secure, encrypted texting and telehealth platform [23].

The training consisted of low-cost, guideline-based antihyperglycemics, antihypertensives, and antihyperlipidemics, e.g., metformin, sulfonylureas, pioglitazone, insulin, ACE inhibitors, amlodipine, statins [24]. Training also included patient-, physician-, and healthcare system-related barriers to obtaining medications [6,24,25,26,27,28]. The training focused on adverse events and side effects months 1–6 and dosing, titration, and emergencies months 7–12. A bilingual physician who was also a Texas-certified CHW-instructor provided the weekly training.

We placed several measures to ensure patient safety. We provided glucometers with supplies to participants. All participants were instructed to measure glucose daily at various times or more if clinically indicated. At each group visit, we distributed a handout for participants to record their glucose levels during the month that also included safe glucose parameters (fasting, pre-meal, post-prandial) and severe hypo- or hyperglycemic values for which they would need to seek immediate attention. Study physicians asked participants for this information at each visit and CHWs did the same during weekly (months 1–6) bimonthly (months 7–12) mHealth communication. Participants who exhibited controlled HbA1c levels without hypo- or hyperglycemia on home logs were down-titrated in frequency of home glucose checks e.g., 1–3 times weekly. Participants who were newly initiated on injectables or needed ongoing assistance received a 1:1 appointment with a pharmacy educator.

### 2.2. Measures

Clinical outcomes included change of HbA1c (primary), blood pressure, and BMI from baseline to six months, which was the duration of the group visits. These outcomes were obtained at the last group visits. They were not gathered when they returned to clinic after month 6, e.g., months 9 and 12 due to the introduction of other variables including loss of clinic or medication eligibility, receiving care from new providers, etc. Detailed outlines of obtaining clinical outcomes are consistent with our prior work, e.g., blood pressure measured with loose clothing, arm at 90 degree [19].

We measured change of CHW knowledge with pre/posttests: TEST-1 (n = 20 questions) given at baseline and month 6, TEST-2 (n = 19 questions) given at month 7 and month 12. Tests were multiple choice with 3–5 answer options, original to this study, and based on current practices (Appendix A and Appendix B) [24]. TEST-1 addressed adverse events and side effects. TEST-2 included medication dosing, titration, and emergencies.

Evidence of CHW training application was measured by the identification of patient-, provider-, healthcare system-related medication barriers as reflected on weekly (months 1–6) and bimonthly (months 7–12) mHealth reports. CHWs recorded the details of their participant conversations that averaged 5–10 min and sent these reports to study physicians. Information was deidentified with a unique participant study number.

### 2.3. Statistical Analysis

Statistical analysis was performed in Sigma Plot 13.0. A two tailed paired *t*-test was performed for change of HbA1c (primary outcome), blood pressure, weight, BMI, and the pre-/post-tests from baseline to six months. We used intention-to-treat analysis. Missing clinical data were handled as last observation carried forward: HbA1c (n = 4), BMI (n = 0), and systolic (n = 1) and diastolic (n = 1) blood pressure. Missing answers for all CHW tests combined were TEST-1 (0.8%, n = 2 questions) or TEST-2 (0.4%, n = 1 question) and omitted from the analysis. Secondary outcomes failed test for normality (Shapiro–Wilk test) and, therefore, Wilcoxon Signed Rank test was used for these comparisons. Statistical significance was defined as *p* < 0.05. 

## 3. Results

Participant (n = 55) and CHW (n = 6) baseline characteristics are illustrated in Table 1 and Table 2, respectively. Individuals had several similarities including slightly more females, middle age, employment type, and religious affiliation. Participants averaged 11.5 years since the time of diabetes diagnosis, elevated HbA1c (8.98% (74 mmol/mol)) and BMI (33.8 kg/m^2^) levels, and controlled blood pressure (131.5/76.3 mmHg). Most participants were prescribed oral hypoglycemics without injectables (65.5%), followed by orals with injectables (32.7%) and solely lifestyle modifications (1.8%). All CHWs finished high school and had full- or part-time work or student obligations outside of the current study. Most were bilingual (83.3%) and born in Central America (66.7%). CHWs lived in communities that were low-income (median USD 47,545), high in poverty (19.3%), largely Latino(a) (47.7%), and below-average high school graduation rates (76.7%).

### 3.1. Clinical Outcomes

Participants’ HbA1c levels significantly improved (9.0% (75 mmol/mol) to 7.8% (62 mmol/mol)) from baseline to six months (*p* = 0.001). Mean blood pressure levels remained controlled (systolic 131.5 to 130.0 (*p* = 0.622); diastolic: 76.3 to 75.0 (*p* = 0.986) and BMI unchanged (33.8 to 33.9 kg/m^2^; *p* = 0.347).

### 3.2. Community Health Worker Outcomes

**Figure 1:**
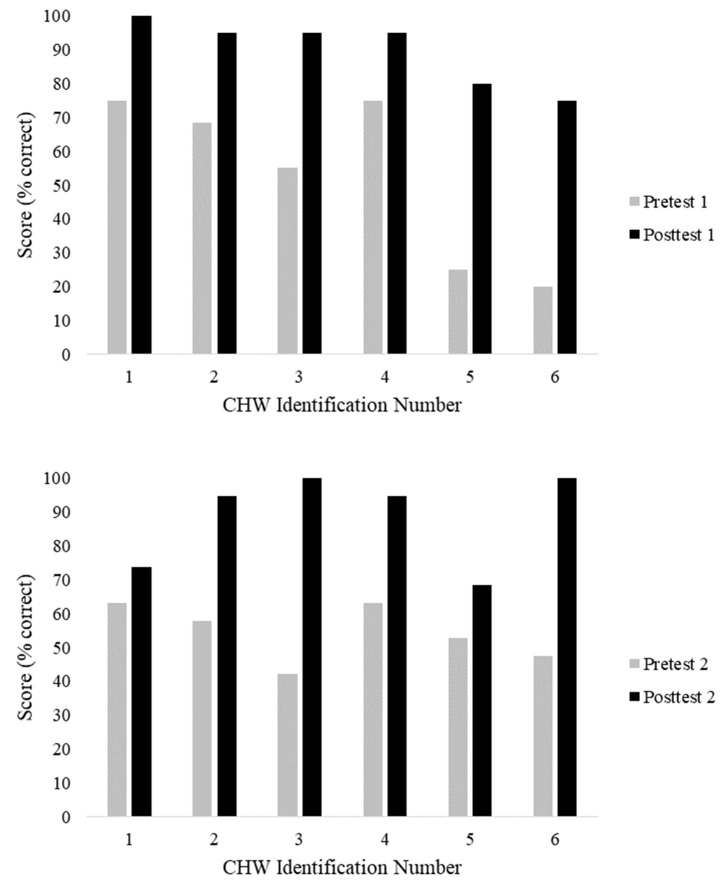
(**Top**) (*p* = 0.002) and TEST-2 (**Bottom**) (*p* = 0.0019). CHW: Community Health Worker

The results of TEST-1 and TEST-2 revealed significant improvements from pre- to post-test (Figure 1), and all CHWs improved their scores on both tests. On TEST-1, CHWs averaged 10.5/20.0 (range 4–15) on the pre-test and 18.2/20.0 (range 15–20) on the post-test (*p* = 0.002). On TEST-2, CHWs averaged 10.3/19.0 (range 8–12) on the pre-test and 17.3/19.0 (range 13–19) on the post-test (*p* = 0.0019).

The results of the 12-month mHealth data are summarized in Table 3. There was strong evidence of CHWs applying their training in their work. A total of 984 incidences were recorded. The highest number of barriers were at the patient-level (n = 610), followed by the healthcare system (n = 223), and provider (n = 151). Of the patient-level barriers, glucose level education was the most common (n = 367) followed by adherence strategies (n = 195). Of the provider-level barriers, medication identification, use, and instructions occurred the most (n = 93), and of the healthcare system, obtaining refills and correct amounts from the pharmacy was most frequent (n = 105).

## 4. Discussion

Preventing diabetes sequelae in uninsured individuals is critical as they are greatly limited in ongoing therapeutic options, e.g., dialysis, sustaining employment thereafter is dismal. This study revealed promising participant and CHW outcomes associated with a telehealth-based CHW medication training program that have not been demonstrated in prior studies. Specifically, participants reduced HbA1c levels (−1.2%, *p* = 0.001) and CHWs improved knowledge at both 6 (*p* = 0.002) and 12 months (*p* = 0.0019). CHWs also demonstrated application of their training by identifying nearly 1000 medication barriers. An unchanged BMI may further support increased medication adherence. Though participants sought weight reduction during the diabetes program, they expressed concern with hypoglycemic agents hindering this process, which is common with several low-cost hypoglycemics (sulfonylureas and pioglitazone). Blood pressure did not change significantly, which was likely a reflection of well-controlled baseline levels. These findings demonstrate a personalized, culturally sensitive, and accessible modality to improve patient outcomes and prevent long-term sequelae.

Prior investigations have also demonstrated the value of improving CHW knowledge, though patient health outcome information is limited. A systematic review (n = 122) revealed that CHW training resulted in improved knowledge, performance, job satisfaction, and community confidence and that ongoing training with back-up support was needed to avoid post-training competency loss [30].

We previously showed the ability to train CHWs via telehealth [13]. We also demonstrated the value of a multifaceted diabetes program on patient outcomes [19]. However prior to this study, investigators had not reported on the ability to train CHWs on diabetes medications and the effect of this intervention on patient outcome. Studies have underscored the importance of ongoing support and training, rather than reliance on initial, one-time training, in the success of CHW-led interventions [13,18]. This study is consistent with these findings and also provides valuable patient outcome data.

The training CHWs received was instrumental in providing a personalized approach to care by tailoring patient-specific interventions [16,17]. Specifically, this training empowered CHWs to address adherence barriers at patient-, provider-, and healthcare system-levels. The frequency of CHW-participant contact may have contributed to the outcomes (identification of abnormal blood glucose, medication adherence issues, etc.). For example, CHWs assisted in patient-related barriers by providing education regarding glucose levels, adherence strategies, and correct dosing or titration. This was particularly important as nearly all (98.2%) participants were prescribed antihyperglycemic medications. One-third of participants were prescribed injectables, which have been associated with nonadherence [4]. At the study start, we observed that participants often did not know normal fasting and non-fasting glucose levels and tended to self-titrate or not take medications based on their beliefs. CHWs frequently reviewed normal levels, giving individuals ownership of their disease and decreasing fear of hypoglycemia, which has shown to have strong associations with nonadherence [4]. Further, since adverse events are a strong predictor of nonadherence [5], a substantial amount of training (six months) was devoted to this topic. We did not experience CHWs “guessing” or providing misinformation to participants, which was an important risk to consider. We attributed this to their ongoing, weekly training that included physician support and feedback.

In addition, CHWs addressed provider-barriers. CHW training included case studies on medications, strengths, and dosing, which enabled them to communicate with participants and physicians, eliciting key information to decrease barriers. Providing CHWs with updated participant medication lists allowed the ability to proactively gather potential adverse event information. A critical part of CHW training was medication eligibility and cost. Some participants required paperwork for costly medications. Providers may not have been aware of low-cost medications or how to inform individuals to obtain high-cost drugs. CHWs informed the study physician of an individual’s needs and linked participants to coupons and pharmacies to obtain affordable drugs. Further, individuals were often nonadherent at work due to coordinating breaks, food, and medication timing. CHWs asked the physician for potential changes, such as metformin IR (twice daily) to metformin XL (daily) and discussed adherence strategies with participants (e.g., phone alarm, simple meal suggestions).

Further, CHWs assisted in healthcare system-level barriers by assisting with refills, appointments, and clinic eligibility. They taught participants how to read medication bottle labels including the number of refills and expiration. This assisted participants in understanding how to renew medications on time. CHWs helped participants navigate obtaining refills including receiving the correct number of pills (30- versus 90-day supply) to decrease frequent transportation needs. CHWs also taught participants how to schedule appointments and obtain clinic eligibility (if needed) to reduce gaps in care.

Providing CHW training via telehealth was a key in ongoing communication. All CHWs had other part- or full-time jobs and stated that weekly in-person training would not be feasible. Though concerns of transportation time are typically linked with rural environments, this study’s urban setting (Houston, TX) created major barriers to meeting together, as CHWs lived in various areas of the city and traffic significantly impeded commuting. This study aligns with prior work showing the value of telehealth training, though studies related to CHWs are limited [13,31,32].

The study is limited by sample size and occurred in one setting, hindering generalizability. Another limitation was the challenge of measuring medication adherence. We considered measuring adherence but found modalities problematic in our population. These include implausibility of patient recall and the use of multiple pharmacies in multiple systems, limiting refill history [6,19,33]. Similarly, pill counting was not reliable (e.g., duplicate medications from different pharmacies). Therefore, we considered clinical outcomes, specifically HbA1c, the most predictable measure of adherence in our study.

## 5. Conclusions

This study highlighted that a telehealth-based diabetes medication training for CHWs allowed for a personalized approach to identify barriers to care at several levels. The intervention was associated with improved participant glycemic control and CHW knowledge and is a promising modality to equip CHWs to address multifaceted barriers to care and adherence. To fully understand the impact on patient outcomes, multicenter longitudinal evaluations are warranted.

## Figures and Tables

**Table 1:** Baseline Participant Characteristics (n = 55).

	**n (%) or Mean (SD)**
**Sex**	
Female	36 (65.5%)
**Age (years)**	52.9 (±8.2)
Range	27–80
**Work**	
Construction/landscaping	17 (30.9%)
Domestic	26 (47.3%)
Food Service	5 (9.1%)
Unknown	7 (12.7%)
**Religious Affiliation**	
Christianity	54 (98.2%)
Unknown	1 (1.8%)
**Time since diabetes diagnosis (years)**	11.5 (±8.0)
Range	0.5–28
**HbA1c (%)**	8.98 * (±2.4)
Range	5.7–14.9 **
**Antihyperglycemic Treatment**	
Lifestyle only	1 (1.8%)
Oral only	36 (65.5%)
Orals + injectables	18 (32.7%)
Injectables only	0 (0.0%)
**Blood Pressure (mmHg)**	
Systolic	131.5 (±17.2)
Range	100–175
Diastolic	76.3 (±9.4)
Range	56–111
**Body Mass Index (kg/m^2^)**	33.8 (±8.0)
Range	23.2–56.9

* 74 mmol/mol, ** 39–139 mmol/mol.

**Table 2:** Baseline Community Health Worker Characteristics (n = 6).

	**n (%) or Mean (SD)**
**Sex**	
Female	4 (66.7%)
**Age (years)**	48.3 (±10.37)
**Work outside of current study**	
Construction	1 (16.7%)
Domestic	1 (16.7%)
Ministerial/pastoral	2 (33.3%)
Student	1 (16.7%)
**Religious Affiliation**	
Christianity	6 (100%)
**Education (completed)**	
High school	6 (100%)
Junior college	1 (16.7%)
Completed college	2 (33.3%)
**Language**	
Bilingual (English/Spanish)	5 (83.3%)
Spanish only	1 (16.7%)
**Birth origin**	
United States (Texas)	2 (33.33%)
International	
El Salvador	1 (16.7%)
Guatemala	1 (16.7%)
Mexico	2 (33.3%)
Residential Demographics *	
Median Income ($)	47,545 (±12,297.44)
Below federal poverty level	19.3 (8.19%)
High school graduates	76.7 (7.14%)
Latino(a)	47.7 (11.4%)

* United States Census Bureau data by zip code [29].

**Table 3:** Community Health Worker–participant weekly telehealth report data, stratified into patient-, provider-, and healthcare-system-level barriers [6].

**Barrier-Level**	Incidence (n) *
**Patient**	
Glucose level education	367
Adherence education	195
Correct dosing, titration	48
**Provider**	
Medication identification, use, and instructions	93
Medication side effects, adverse events, emergencies	34
Medication eligibility, cost	24
**Healthcare system**	
Obtaining refills; Correct dosing/amount from pharmacy	105
Clinic appointments	81
Clinic eligibility	37
**Total**	**984**

* CHW–participant interaction occurred during three, 12-month diabetes group visit cohorts consisting of 6-month group visits and 6-month follow-up with bimonthly CHW call/text with care in clinic (n = 55 participants).

## Appendix A. Community Health Worker TEST-1 and TEST-2. Correct Answers Are Bolded

TEST-1

1. The most common side effect of metformin is:
  - Weight loss
  - Weight gain
  - **Stomach upset**
  - Low blood sugar
2. What diabetes medication(s) are FOUR dollars at CVS or Walmart?
  - Actos
  - Metformin
  - The “g” drugs e.g., glimepiride, glyburide, glipizide
  - **B and C**
  - All of the above
3. What medication class end in “pril” or “artan” i.e., lisinopril, losartan?
  - Cholesterol medications
  - Diabetes medications
  - Weight loss medications
  - **Blood Pressure medications**
4. The most common side effect of the “prils”is:
  - **Cough**
  - Diarrhea
  - Stomach upset
  - Leg cramps
5. What medication class end in “statin”?
  - **Cholesterol medications**
  - Diabetes medications
  - Weight loss medications
  - Blood Pressure medications
6. The most common side effect of the “statins” is:
  - **Leg cramps**
  - Stomach upset
  - Weight loss
  - Cough
7. A concerning side effect of Actos (pioglitazone) is:
  - A rash
  - Headache
  - Blurry vision
  - **Leg swelling**
8. Actos was once taken off the market in other countries since some thought it increased the chances of:
  - Skin infection
  - **Bladder cancer**
  - Amputation
  - Kidney failure
9. The most common medication used for diabetes care is:
  - The “g” drugs e.g., glimepiride, glyburide, glipizide
  - Actos (pioglitazone)
  - **Metformin**
  - Insulin
10. What is NOT common with patients taking metformin?
  - Weight loss
  - Stomach upset
  - Diarrhea
  - **Swelling of the legs**
11. What does insulin do to blood sugar?
  - Increases blood sugar
  - Does not change blood sugar
  - **Decreases blood sugar**
12. What does orange juice do to blood sugar?
  - **Increases blood sugar**
  - Does not change blood sugar
  - Decreases blood sugar
13. What US pharmacy sells some insulin for $25 per bottle?
  - CVS
  - **Walmart**
  - Kroger
  - Walgreens
14. What cholesterol pill is the strongest to decrease triglycerides?
  - Simvastatin
  - Glimepiride
  - Lisinopril
  - **Fenofibrate**
15. What is the maximum dose of metformin per day?
  - 500
  - 1000
  - 1500
  - **2000**
16. What medication(s) is often prescribed after a heart attack and/or stroke?
  - A “statin”
  - Aspirin
  - Plavix
  - **All of the above**
17. Which medication(s) may cause patients to GAIN weight?
  - Actos/pioglitazone
  - The “g” drugs e.g., glimepiride, glyburide, glipizide
  - Metformin
  - Insulin
  - **A, B, and D**
18. Which medication should be stored in the refrigerator?
  - **Insulin**
  - Enalapril
  - Metformin
  - Actos/pioglitazone
  - the “g” drugs e.g., glimepiride, glyburide, glipizide
19. Which medication must be taken with food?
  - Atorvastatin
  - Lisinopril
  - **The “g” drugs e.g., glimepiride, glyburide, glipizide**
  - Actos/pioglitazone
20. Which is NOT correct?
  - Novolog insulin works fast (short-acting)
  - **Humalog insulin works “in the middle” (intermediate)**
  - NPH insulin works “in the middle” (intermediate)
  - Lantus insulin works for a long time (long-acting)

## Appendix B. Community Health Worker TEST-1 and TEST-2. Correct Answers Are Bolded

TEST-2

1. How often should patients take metformin “IR”?
  - Once daily
  - **Twice daily**
  - Three times daily
  - Four times daily
2. How often should patients take metformin “XL”?
  - **Once daily**
  - Twice daily
  - Three times daily
  - Four times daily
3. How is metformin IR started?
  - 250mg daily
  - **500mg daily**
  - 1000mg daily
  - 1500mg daily
4. How much is metformin IR/XL increased each week?
  - 250mg
  - **500mg**
  - 1500mg
  - 2000mg
5. What medication is the most effective to decrease blood sugar?
  - Metformin(a) IR
  - Metformin(a) XL
  - **They are the same**
6. Less than ______ is “severe” low blood sugar.
  - 20
  - 30
  - 40
  - **50**
7. A patient’s blood sugar is 62. He drank a cup of orange juice. After how many minutes should he recheck his blood sugar?
  - 5
  - **15**
  - 45
  - 60
  - 90
8. A patient’s medication is expired. Now, the medication may:
  - Be stronger than before
  - Be weaker than before
  - Not change at all
  - **All of the above**
9. What is NOT a symptom of high blood sugar?
  - Increased urination
  - Increased thirst
  - **Increased constipation**
  - Increased hunger
10. Your patient tells you that her blood sugar is 425. What should you do?
  - Tell her to drink more non-sugary liquids
  - Tell her to recheck it and make sure the number is right
  - Ask her if she took her medications as prescribed today
  - Tell her to continue monitoring until it goes down and if not, call her doctor
  - **All of the above**
11. What is “sliding scale insulin”?
  - A type of short-acting insulin
  - A type of long-acting insulin
  - **Taking a certain amount of insulin based on blood sugar**
  - Decreasing the amount of insulin the patient takes over time
12. What is a reasonable way to increase the amount of insulin used if fasting and afternoon/evening sugars are high?
  - **Increased by 3 units every 3 days**
  - Increased by 5 units every day
  - Increase by 10 units every 10 days
  - Increase by 7 units every day
13. Where is the preferred place to inject insulin?
  - Upper arm
  - **Stomach**
  - Thigh
  - Buttocks
14. What is the starting dose for glimepiride?
  - **2 mg/day**
  - 4 mg/day
  - 5 mg/day
  - 15 mg/day
15. What is the maximum dose for glimepiride?
  - 2 mg/day
  - 5 mg/day
  - **8 mg/day**
  - 45 mg/day
16. What is the starting dose for Actos?
  - 2 mg/day
  - 4 mg/day
  - 5 mg/day
  - **15 mg/day**
17. What is the maximum dose for Actos?
  - 2 mg/day
  - 5 mg/day
  - 8 mg/day
  - **45 mg/day**
18. What medication can NOT be started in kidney failure (end stage)?
  - Glimepiride
  - Insulin
  - Actos
  - **Metformin**
19. A patient’s bottle of medicine is empty. She has 2 refills, expiration 1/1/2019. What should she do?
  - **Call the clinic (pharmacy) to fill for refills**
  - Call the clinic to make an appointment with her doctor
  - Wait until her appointment next month and ask her doctor.
